# Case Report: Use of novel AR registration system for presurgical planning during vestibular schwannoma resection surgery

**DOI:** 10.3389/fsurg.2024.1304039

**Published:** 2024-03-04

**Authors:** Joshua Olexa, Annie Trang, Rebecca Flessner, Mohamed Labib

**Affiliations:** Department of Neurosurgery, University of Maryland School of Medicine, Baltimore, MD, United States

**Keywords:** augmented reality, vestibular schwannoma, presurgical planning, mixed reality, navigation

## Abstract

**Background and importance:**

Vestibular schwannomas are benign tumors and are the most common tumor found in the cerebellopontine angle. Surgical management of these lesions involves consideration of various operative approaches, which can have profound effects on procedural course and patient outcomes. Therefore, a comprehensive understanding of the location of the tumor and surrounding anatomical structures is vital for a positive outcome. We present a case of a 47-year-old female patient with vestibular schwannoma. A novel mixed reality (MR) system was used to register patient-specific 3D models onto the patient's head for operative planning and anatomical visualization.

**Case description:**

A 47-year-old female presented with a history of left-sided hearing loss, tinnitus, and episodic left facial tingling. Magnetic Resonance Imaging (MRI) demonstrated a 3.3 cm enhancing lesion in the left cerebellopontine angle at the with mass effect on the brachium pontis and medulla. Surgical resection was performed via retrosigmoid craniotomy.

**Conclusions:**

In this study, we report the use of Augmented Reality (AR) visualization for planning of vestibular schwannoma resection. This technology allows for efficient and accurate registration of a patient's 3D anatomical model onto their head while positioned in the operating room. This system is a powerful tool for operative planning as it allows the surgeon to visualize critical anatomical structures where they lie on the patient's head. The present case demonstrates the value and use of AR for operative planning of complex cranial lesions.

## Introduction

Vestibular schwannomas are benign tumors and are the most common tumor found in the cerebellopontine angle (1). Symptoms of vestibular schwannoma include sensorineural hearing loss, tinnitus, unsteadiness and less commonly may include vertigo, facial pain, numbness and weakness (2). The goal of vestibular schwannoma management is to reduce mass effect and preserve nerve function (3). The major treatment options for vestibular schwannomas are surgery, radiation therapy and observation (3). When surgery is indicated, various approaches can be performed such as translabyrinthine, retrosigmoid and middle fossa craniotomy (2). Each of these approaches involve critical neuroanatomy that require delicate management to optimize functional outcomes and reduce complications. Complications of surgical removal of vestibular schwannomas can include CSF leak, headache and worsened or persistent neurologic deficit including cranial neuropathies (3). In order to minimize these complications, surgeons must have a good understanding of the tumor's location and surrounding structures. Improvements in MRI imaging have allowed for the ability to visualize the relationship of these tumors with surrounding neurovascular structures for more efficient and targeted treatment plans (1). Although MRI provides a great deal of information about the anatomy, traditional surgical planning with 2D MRI-based imaging can be limited in providing a complete understanding of the tumor and its surroundings in 3D space.

 Augmented Reality (AR) is a technology that can superimpose 3D virtual models onto the user's real-world environment. AR has the potential to improve the accuracy and effectiveness of surgical planning by providing surgeons with a 3D view of the tumor and its surrounding structures that can be used to plan the ideal surgical approach. AR provides the benefits of both 3D visualization, and the ability to visualize these models superimposed onto the real environment and patient. The purpose of this study is to assess the benefit of a novel AR registration system for operative planning in the setting of a retrosigmoid craniotomy for resection of a vestibular schwannoma.

## Case description

**Figure 1:**
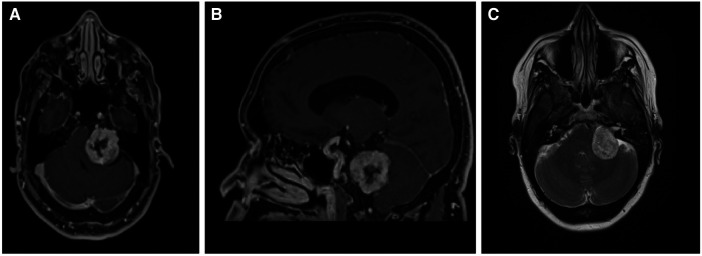
Diagnostic MRI study demonstrating the vestibular schwannoma (**A**) axial T1 with contrast; (**B**) sagittal T1 with contrast (**C**) axial T2.

A 47-year-old female presented for neurosurgical consultation with a history of hearing loss, tinnitus, and episodic left facial tingling. Magnetic Resonance Imaging (MRI) demonstrated a 3.3 cm contrast-enhancing left cerebellopontine angle (CPA) lesion with notable mass effect on the brachium pontis and medulla (Figure 1). Management strategies were discussed with patient, and plan was made for surgical resection via retrosigmoid craniotomy approach.

## Segmenting anatomical structures

**Figure 2:**
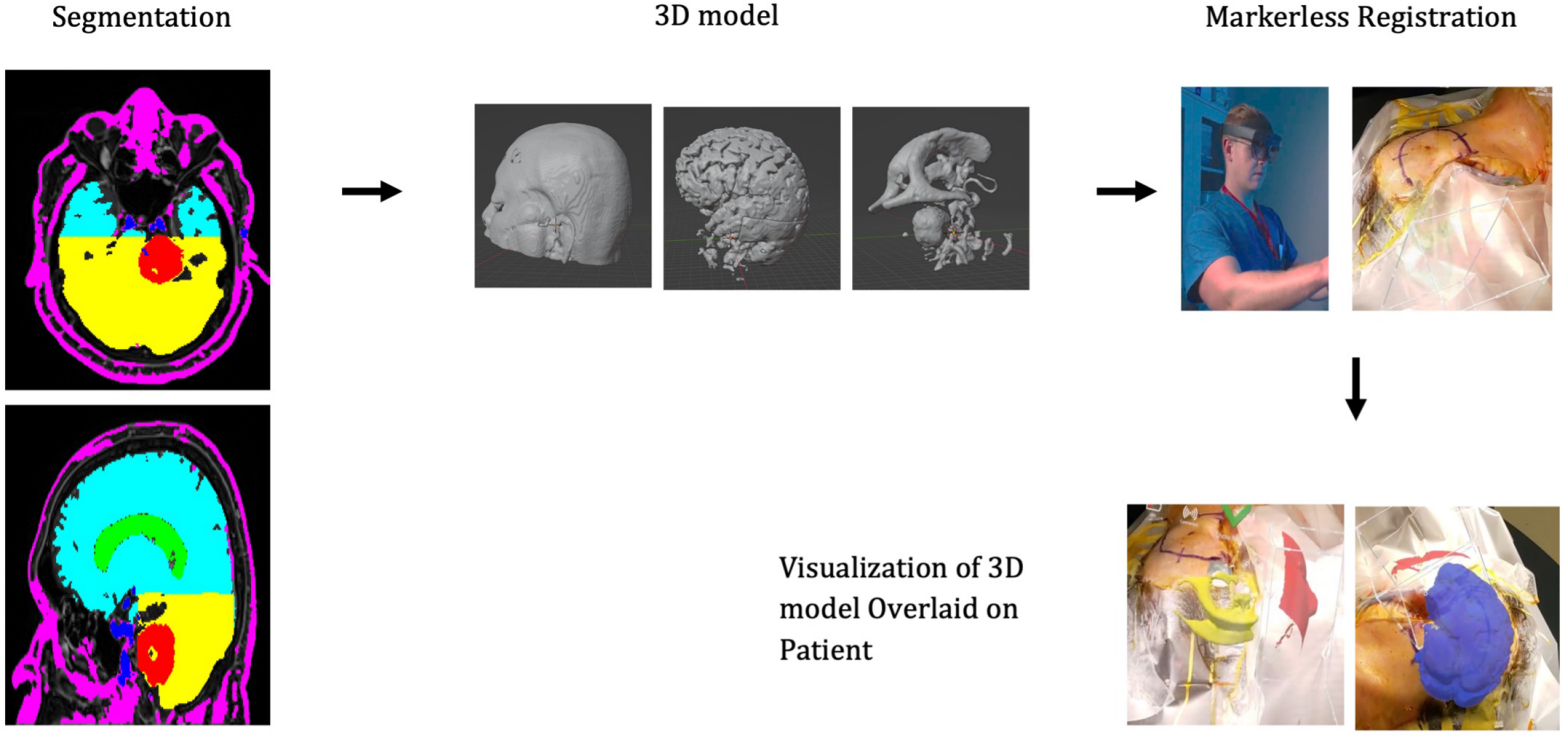
Workflow of the AR technology platform. The patient anatomy is first segmented and converted into a 3D model. The model is loaded onto the AR headset. While wearing the headset, the surgeon scans the patients’ head. Registration algorithms are processed on the headset and the 3D model can be visualized aligned/overlaid with the patients’ head.

3D models were generated based on the patient's preoperative MRI scan. Anatomy of interest—tumor, ventricles, vasculature, and whole brain—were manually segmented using ITKsnap. Figure 2 demonstrates the segmented MRI with schwannoma shown in red, ventricles in green, whole brain in turquoise/yellow, and vessels in blue. The patient's face was segmented and reconstructed for registration via auto segmentation algorithms (Hoth Intelligence Inc, Philadelphia, Pennsylvania). All segmented structures were merged and converted into a 3D digital model. The digital models underwent basic processing to reduce the file size to optimize performance, visual quality, and usability for an AR head-mounted display (HMD).

## AR technology platform

The AR application was developed by Hoth Intelligence (Philadelphia, Pennsylvania) to operate on the Microsoft Hololens 2 HMD (Redmond, Washington). The Microsoft Hololens 2 is an untethered optical see-through head-mounted display that superimposes virtual content (i.e., holograms, images, screens) onto the users' real-world field of view. A clinical workflow of the AR system is described in Figure 2.

## 3D model registration to patient

The application used in this study superimposes 3D digital models onto the head using an ultra-fast, fiducial-less registration process. Information from the headset sensors (depth sensor, RBG camera, and stereosensor) are merged with coordinate data from preoperative MRI scan to align the 3D model to the patient's head. After the patient was pinned in the operative position, the patient-specific 3D model was registered onto the head via the AR headset.

### Mixed reality viewing

**Figure 3:**
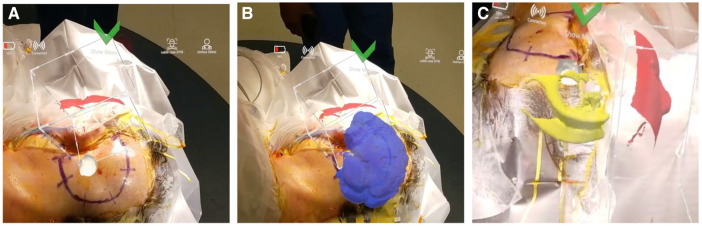
Surgeons’ view through the AR headset displaying 3D model overlaid on patients’ head. (**A**) Visualization of the tumor in white. (**B**) Whole brain shown in blue. (**C**) Ventricles in yellow.

Once registered, the surgeon was able to freely move around the patient to visualize the 3D model overlaid onto the patients' head (Figure 3 and Supplementary Video S1). The added AR visualization allowed the surgeon to confirm proper approach and incision/craniotomy locations. Additionally, the ability to hide and show different layers of the model allowed the surgeons to discuss other operative considerations such as nearby vascular structures surrounding the lesion and proximity to brainstem.

## Surgical strategy

To relieve brainstem compression, resection with a retrosigmoid craniectomy was performed. With the help of neuro-navigation, the sigmoid sinus and transverse sigmoid junction were identified and skeletonized. Cisterna magna was opened to decompress the cerebellum and access both the CPA and internal auditory canal (IAC) porus. The tumor was identified caudally above the jugular foramen and debulked. The posterior fossa dura was further incised to access the IAC, which revealed intact tumor from the most distal aspect of the canal into the CPA. To preserve nerve function, a small segment of tumor overlying the facial nerve was left in place before duraplasty and cranioplasty. Postoperatively, facial numbness resolved, and the patient was otherwise neurologically stable.

## Discussion

We describe a novel use of AR for use in surgical planning for retrosigmoid craniotomy for resection of a cerebellopontine angle lesion. The use of virtual reality (VR) for surgical planning in neurosurgery has been shown to increase understanding of complex anatomic relationships to improve surgical planning (4). AR allows for even further advancement in surgical planning by integrating 3D visualization onto the real-world environment. We describe our experience using AR to overlay patient-specific 3D models onto the patient's head with a marker-less registration system. Previous studies have shown AR to be effective for both surgical planning and intraoperative guidance using standard patient registration methods (5). These standard registration methods are time consuming and require expensive systems that have a large amount of hardware. This novel technology allows for the benefits of increased visualization and understanding of patient specific anatomy with an efficient, marker-less registration system. Use of the AR system allowed the surgeon to plan surgical approaches such as incision and craniotomy location as well as discuss the spatial relationship between the lesion and other anatomical structures.

The use of AR for visualizing and overlaying patient anatomy has been shown to be an effective surgical planning tool (6). Reports have described the use of AR for craniotomy and trajectory planning using pre-operative MRI of cranial meningiomas. Additionally, other reports have described the use of AR for navigation during procedures such as needle biopsy and external ventricular drain placement. AR systems used in these other reports typically utilize fiducial markers and/or require multiple camera systems to register anatomy with the patients (7–9). This is a case report showing the use of a novel markerless registration AR system for visualization of patient-specific anatomy overlaid onto the patient in the operating room for surgical planning. The system does not require fiducial markers and operates entirely our of the headset without the need for external camera systems. For this case, while wearing an augmented reality headset, the surgeon was able to overlay a 3D model of the patients' vestibular schwanomma and brain onto the patients' head in the proper location and orientation. This allowed the surgeon to plan the craniotomy location and optimize proper head position. As such the system serves as an effective planning tool prior to making an incision. Right now the system—given its size and speed—serves as an effective adjunct to traditional neuronavigation however, in the future this technology has the potential to serve as a primary image guidance system. Further study of this technology is needed to characterize its benefits and accuracy compared with traditional surgical planning approaches.

## Conclusion

In this study, we report the use of AR visualization for planning of retrosigmoid craniotomy for removal of a vestibular schwannoma. This technology is advantageous in surgical planning as it allows for enhanced visualization with a fast, user-friendly setup. Future studies are needed to further characterize the accuracy of this novel technology and benefits such as faster setup time, operative time, improved surgical outcomes and possible cost savings.

## Data Availability

The original contributions presented in the study are included in the article/**Supplementary Materials**, further inquiries can be directed to JO, jolexa@som.umaryland.edu.

## Supplementary material

Supplementary Video S1 Footage of 3D model overlaid onto patients' head as viewed through the headset.
